# Monoclonal antibody 4C5 prevents activation of MMP2 and MMP9 by disrupting their interaction with extracellular HSP90 and inhibits formation of metastatic breast cancer cell deposits

**DOI:** 10.1186/1471-2121-11-51

**Published:** 2010-07-05

**Authors:** Dimitris Stellas, Avraam El Hamidieh, Evangelia Patsavoudi

**Affiliations:** 1 Department of Biochemistry, Hellenic Pasteur Institute, 127 Vasilissis Sofias Ave., 11521 Athens, Greece; 2 Department of Medical Instrumentation, Technological Educational Institution of Athens, Ag. Spyridonos str., Egaleo, 12210 Athens, Greece

## Abstract

**Background:**

Heat shock protein 90 (HSP90) is a molecular chaperone that is considered a new target for the treatment of cancer. Increasing data reveal an extracellular chaperoning activity for HSP90. Here we investigate the interaction of the secreted isoforms of HSP90 with matrix metalloproteinases (MMP) MMP2 and MMP9. Moreover we examine the role of a monoclonal antibody (mAb) against HSP90, mAb 4C5, regarding these interactions and its value as a potential inhibitor of human breast cancer cell invasion and metastasis.

**Results:**

Our results showed that both HSP90α and HSP90β are secreted by MDAMB453 human breast cancer cells and interact with MMP2 and MMP9. MAb 4C5, while not affecting the secretion of inactive MMPs, inhibits their activation by disrupting their interaction extracellularly with both isoforms of HSP90. The *in vivo *studies revealed that mAb 4C5 significantly inhibits the metastatic deposit formation of MDAMB453 cells into the lungs of SCID mice.

**Conclusion:**

Both isoforms of HSP90 are secreted by MDAMB453 cells and interact with MMP2 and MMP9. MAb 4C5 prevents MMP2 and MMP9 activation, by disrupting their interaction with HSP90. Finally mAb 4C5 significantly inhibits the metastatic deposit formation of MDAMB453 cells, by preventing their extravasation and infiltration in the lung tissue and therefore it could be used as a potential therapeutic agent for cancer metastasis.

## Background

The dissemination of tumor cells from their primary site of growth to distant organs is the major cause of morbidity and death among cancer patients [1,2]. Thus, inhibition of invasion and metastasis of cancer cells is of great importance in the treatment of cancer. Cancer cell invasion and metastasis is considered to be a complex, multi-step process, during which malignant cells detach from their point of origin, migrate and invade surrounding tissues, enter the vasculature, circulate and reach secondary sites, extravasate and establish metastatic foci [3,4].

One well-characterized property of invasive tumors is their ability to accelerate the degradation of the extracellular matrix, by matrix metalloproteinases (MMPs) [5].This degradation provides access to the vasculature and lymphatic system, allowing tumor dissemination. MMPs have increased expression and activation in almost all human cancers[6]. More specifically, MMP2 and MMP9 are of particular interest because in addition to gelatin they degrade type IV collagen, the basic component of the basement membrane, which is the main barrier separating *in situ *and invasive carcinoma [7,8].

The heat shock protein 90 (HSP90) is a molecular chaperone which exists in mammalian cells in two isoforms that share 86% aminoacid conservation (HSP90α and HSP90β). It is one of the most abundant cytoplasmic proteins in unstressed cells, where it performs housekeeping functions, controlling the activity, intracellular disposition and proteolytic turnover of a variety of proteins. Over the past years there has been increasing evidence that HSP90 interacts with a great number of molecules intracellularly, that are involved in the development and/or survival of cancer cells [9-11], allowing mutant proteins to retain or gain function, while permitting cancer cells to tolerate the imbalanced signaling that such oncoproteins create. Recently, we and others have identified a pool of HSP90 at the cell surface, where it was shown to be involved in cancer cell invasion [12]. Additionally, we have reported results showing that a monoclonal antibody (mAb) recognizing both the α and the β isoforms of HSP90, mAb 4C5, inhibits melanoma cell invasion and metastasis by binding selectively to the surface pool of HSP90 [13]. Finally, we have presented data indicating that surface HSP90 interacts specifically with the extracellular domain of HER-2 and that this interaction which is necessary for the receptor's activation leading to breast cancer cell invasion, is disrupted by mAb 4C5 [14].

Taking all the above into consideration together with previous data showing that HSP90α is secreted from fibrosarcoma cells and promotes their invasive capacity through association with MMP2 [15], in the present work we sought to investigate the secretion of both the α and the β isoforms of HSP90 in the conditioned medium of MDAMB453 human breast cancer cells and their possible interaction with MMP2 and/or MMP9. Furthermore and after taking into account our previously mentioned recent data showing that mAb 4C5 inhibits MDAMB453 human breast cancer cell invasion *in vitro *by disrupting the association of cell surface HSP90 with HER-2,[14] in this work we examined: a) the effect of mAb 4C5 on MMP2 and MMP9 secretion and activation b) the ability of this antibody to disrupt the interaction of extracellular HSP90 with MMP2 and/or MMP9 and c) the capacity of mAb 4C5 to inhibit *in vivo*, the formation of metastatic deposits of MDAMB453 cells in the lungs of SCID mice.

## Results

### Both the α and β isoforms of HSP90 are secreted by MDAMB453 breast cancer cells and interact with MMP2 and MMP9

**Figure 1:**
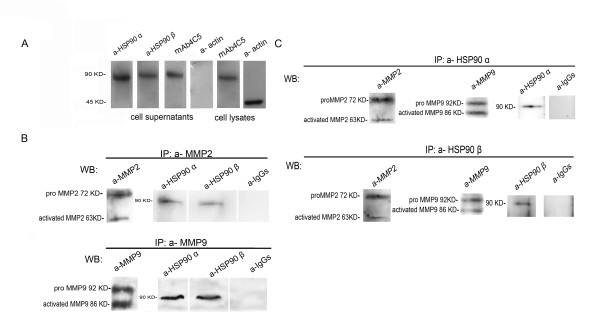
**Both the α and the β isoforms of HSP90 are secreted by MDAMB453 cells and interact with MMP2 and MMP9 metalloproteinases**. (A) Cell lysates were immunoblotted with mAb 4C5 recognizing both isoforms of HSP90 and serving as positive control. Concentrated supernatants derived from the cell cultures as described in Methods, were analyzed by Western blot using anti HSP90 α and anti HSP90 β antibodies and mAb 4C5. Anti beta actin antibody was used in both fractions. Secretion of the two isoforms of HSP90 was detected in the supernatant derived from MDAMB453 cells. The absence of beta actin in the supernatant demonstrates that there is no contamination of this fraction with the intracellular components of cells during the experimental procedure. (B) Western blot analysis of concentrated supernatants derived from MDAMB453 cell cultures, using anti-MMP2 and anti-MMP9 antibodies showed that both the pro-MMP2 and pro-MMP9 as well as their activated forms are present in the culture medium. Proteins derived from the culture supernatant were immunoprecipitated with anti-MMP2 and anti-MMP9 and bound proteins were analyzed by Western blot with anti-HSP90α and anti-HSP90β antibodies. Both isoforms of HSP90 interact with the two metalloproteinases. Irrelevant IgGs and anti-MMP2 or anti-MMP9 antibodies were used as negative and positive controls respectively. (C) Proteins derived from the MDAMB453 cell culture supernatant were immunoprecipitated with anti-HSP90α and anti-HSP90β antibodies and immunoprecipitants were analyzed by Western blot using the anti-MMP2 and anti-MMP9 antibodies. Irrelevant IgGs and anti-HSP90 α or anti-HSP90 β antibodies were used as negative and positive controls respectively. The reverse immunoprecipitation experiment showed that both isoforms of HSP90 interact with pro-MMP2 and pro-MMP9 and to a lesser extent with the activated forms of the two metalloproteinases. IP immunoprecipitation, WB Western blot.

In order to investigate the secretion of the α and β isoforms of HSP90 by MDAMB453 cells, cell lysates and concentrated supernatants derived from the culture of these cells were analyzed by Western blotting using mAb 4C5 which recognizes both isoforms of HSP90[16], and the commercially available anti-HSP90 α and anti-HSP90 β antibodies. As shown in Figure 1A, both the α and the β isoforms of HSP90 are secreted by MDAMB453 cells. The absence of beta actin in the supernatant fraction (Figure.1A) demonstrates that there is no contamination of the culture supernatants with the intracellular components of cells during the experimental procedure. At this point it is important to note that beta actin was never detected in any of the concentrated supernatants used in the experiments described hereafter (data not shown).

An important characteristic of invasive tumors is their capacity to accelerate the degradation of extracellular matrix by MMPs [5]. MMPs are secreted as inactive pro-enzymes and are activated extracellularly by proteolysis [17]. We thus next examined the possible interaction of the secreted pool of HSP90 with MMP2 and MMP9. After confirming the presence of both pro-MMP2 and pro-MMP9 as well as their activated forms in the culture medium of MDAMB453 cells (Figure.1B), we showed by immunoprecipitation experiments in the culture supernatant using anti-MMP2 and anti-MMP9 antibodies followed by Western blot analysis with antibodies against the α and β isoforms of HSP90, that indeed both isoforms of HSP90 interact with the metalloproteinases examined (Figure.1B). In order to further clarify the interaction of extracellular HSP90 with the MMPs studied, reverse co-immunopercipitation experiments were performed. Our results showed that both the α and the β isoforms of HSP90 interact with the inactive pro-MMP2 and pro-MMP9 (Figure.1C). Interaction with the mature forms of the corresponding metalloproteinases was also detected (Figure.1C).

### MAb4C5 prevents MMP2 and MMP9 activation and disrupts their interaction with both the α and the β isoform of extracellular HSP90

**Figure 2:**
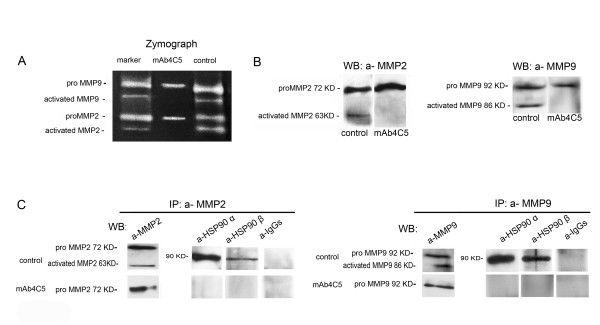
**MAb4C5 prevents MMP2 and MMP9 activation and disrupts their interaction with both the α and the β isoform of extracellular HSP90**. (A) Metalloproteinases derived from the concentrated supernatants of control and mAb 4C5 treated cultures of MDAMB453 cells were isolated and analyzed by zymography, as described in Methods. Proteolysis was detected as a white zone in a dark field. Metalloproteinases previously isolated as described in Methods were used as markers. Activated MMP2 and MMP9 are absent in the mAb 4C5 treated cultures. (B) Proteins derived from the concentrated supernatants of control and mAb 4C5 treated cultures of MDAMB453 cells, prepared as described in Methods, were analyzed by Western blot using anti-MMP2 and anti-MMP9 antibodies. Presence of mAb 4C5 in the culture medium inhibits activation of both metalloproteinases. (C) Proteins from the above described supernatants were immunoprecipitated with anti-MMP2 and anti-MMP9 antibodies and bound proteins were analyzed by Western blot with anti-HSP90α and anti-HSP90β antibodies. MAb 4C5 disrupts the association of both isoforms of HSP90 with the two metalloproteinases, since anti-MMP2 and anti-MMP9 antibodies did not co-immunoprecipitate any detectable levels of HSP90 in the mAb 4C5 treated cultures, when compared with controls. Western blot analysis of the MMP2 and MMP9 immunoprecipitants, using the corresponding antibodies was performed as positive controls and irrelevant IgGs were used as negative controls. IP immunoprecipitation, WB Western blot.

Based on our observation that presence of both the inactive and mature forms of MMP2 and MMP9 were detected in the concentrated supernatants derived from MDAMB453 cell cultures, we next examined the effect of mAb 4C5, the function blocking anti-HSP90 antibody, on the secretion and activation of MMP2 and MMP9. Analysis of concentrated supernatants derived from MDAMB453 cells, with zymography, showed that when 200 μg/ml of mAb 4C5 were included in the culture medium, presence of the inactive pro-enzymes was not affected, while detection of the activated forms of both MMPs was not obtained (Figure.2A). These results were confirmed using antibodies against the metalloproteinases, and western blot analysis (Figure.2B). At this point it is important to note that addition of mAb 4C5 in the culture medium did not affect secretion of HSP90α or HSP90β (data not shown).

Consequently and taking into account the previously demonstrated interactions of both isoforms of HSP90 with MMP2 and MMP9, we explored the role of mAb 4C5 in relation to these interactions, using co-immunoprecipitation experiments. To this purpose, concentrated conditioned media derived either from control or mAb 4C5 treated cultures were immunoprecipitated with antibodies against MMP2 and MMP9 followed by Western blot analysis with antibodies against the α and the β isoforms of HSP90. As shown in Figure 2C, co-immunoprecipitation of both isoforms of HSP90 with the inactive forms of MMP2 and MMP9 was inhibited in the media derived from the cells cultured in the presence of mAb 4C5, when compared with control cultures. This result indicates that mAb 4C5 disrupts extracellularly the interaction of both pro-MMP2 and pro-MMP9 with the two isoforms of HSP90.

### MAb4C5 inhibits the metastatic deposition of MDAMB453 breast cancer cells into the lungs of SCID mice

**Figure 3:**
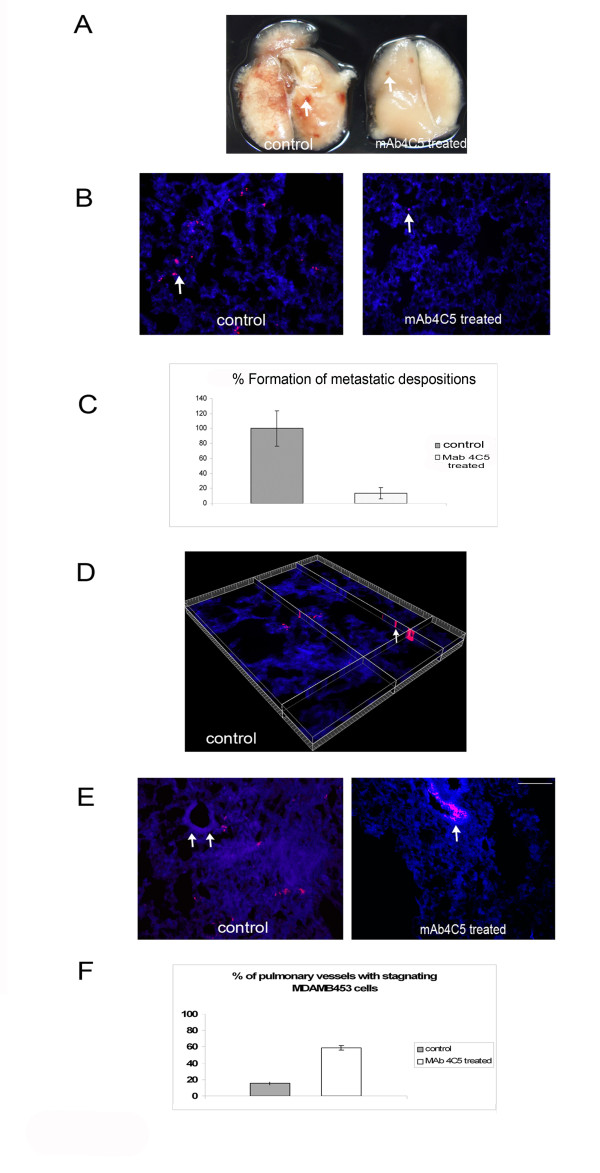
**MAb 4C5 inhibits the metastatic deposition of MDAMB453 cancer cells into the lungs of SCID mice**. MDAMB453 cells were labeled with the fluorescent dye DiI and injected into SCID mice, in the presence either of 100 μg/ml of mAb 4C5 or of an irrelevant antibody (control), as described in Methods. Evaluation of metastatic deposits was performed several hours later. (A) Macroscopic level: An important number of metastatic deposits (arrow) was observed in control animals as compared to mAb 4C5 treated mice. (B) Microscopic level: Representative cryosections of the lungs of control and mAb 4C5 treated mice. The arrows show MDAMB453 cells stained with DiI present in the lung tissue. A significant decrease in the deposition of cancer cells was observed in the mAb4C5 treated mice. (C) Quantitative effect of mAb 4C5 on the metastatic deposition of MDAMB453 cells into the lungs showed an 86.67% inhibition of the metastatic deposition in the mAb 4C5 treated mice when compared to the control animals. (D) A 3D reconstitution of a cryosection from a control animal: The arrow demonstrates infiltration of metastatic deposits in the lung tissue. (E) MDAMB453 cells restrained in the inner surface of large pulmonary vessels derived from mAb 4C5 treated mice. On the contrary, in the lungs from control mice MDAMB453 cells could rarely be detected stagnating in the inner surface of the pulmonary vessels. However, cancer cells were clearly observed dispersed in the lung tissue. Arrows indicate the pulmonary vessels. (F) Quantitative effect of mAb 4C5 on the retention of MDAMB453 cells in the pulmonary vessels. A 15.4% of the vessels visualized in the control animals showed intravascular cancer cell retention. In contrast in the mAb 4C5 treated mice, cancer cells were observed stagnating in the inner surface of 58.76% of the calculated vessels (p < 0.01 Bar, 40 μm).

On the basis of previously reported studies using the MDAMB453 cell line [14] and showing that mAb 4C5 inhibits the invasion of these cells *in vitro*, we next sought to investigate the *in vivo *effect of mAb 4C5 on the metastatic behavior of this breast cancer cell line. Thus, we injected MDAMB453 cells labeled with the fluorescent dye DiI intravenously in SCID mice, either in the presence or in the absence of 100 μg/ml of mAb 4C5. Twenty four hours after the injection, mice were euthanized and the metastatic deposits of MDAMB453 cells were traced and evaluated in the lungs of both the control and the mAb 4C5 treated groups. At the macroscopic level an important number of metastatic cell deposits (arrow in Figure.3A) was observed in control animals as compared to mAb 4C5 treated mice. This was confirmed microscopically where a very important decrease in the deposition of cancer cells was detected in the mAb 4C5 treated mice (arrows in Figure.3B). Quantification of the metastatic deposit formation revealed a 86.67% inhibition in the mAb 4C5 treated mice when compared with control mice (Figure.3C). In order to further visualize the deposition of MDAMB453 cells in the lung tissue, we performed a 3D reconstitution of a cryosection derived from a control animal where the infiltration of metastatic deposits of MDAMB453 cells in the lung tissue is clearly demonstrated (arrow in Figure.3D). It is of interest to note that in the mAb 4C5 treated mice, MDAMB453 cells were often observed stagnating on the inner surface of large pulmonary blood vessels whereas no such images could be detected in the control animals (arrows in Figure.3E). Quantification of this occurrence revealed that in 58.76% of the pulmonary vessels visualized in the, mAb4C5 treated animals, MDAMB453 cells were observed stagnating on the inner surface of the vessels. In contrast, only 15.4% of the vessels observed in the control animals showed intravascular retention of the cancer cells. (Figure 3F).

## Discussion

Our present findings demonstrate that both the α and the β isoforms of HSP90 are secreted by cultured MDAMB453 human breast cancer cells and interact with matrix metalloproteinases, MMP2 and MMP9. Additionally, we show that a monoclonal antibody against HSP90, mAb 4C5, prevents maturation of the two metalloproteinases *in vitro *and inhibits metastatic deposition of breast cancer cells *in vivo*.

We have previously reported the presence of HSP90α and HSP90β on the cell surface of MDAMB453 breast cancer cells [14]. Here we further examined the extracellular localization of HSP90. More specifically, we demonstrated the secretion of both HSP90α and HSP90β in the culture medium of MDAMB453 cells using antibodies specific for the two isoforms. At this point it is important to note that specificity of the anti-HSP90β antibody was further confirmed since, Western blot analysis of human recombinant HSP90α using this antibody gave negative results (see additional files 1 and 2). Our findings, for the first time to our knowledge, reveal secretion of HSP90β by breast cancer cells and are only partly in agreement with existing knowledge. Indeed, previous studies have reported secretion only of HSP90α from various cell types including HT 1080 fibrosarcoma cells [15], human dermal fibroblasts under hypoxia conditions[18] and transforming growth factor alpha stimulated human keratinocytes [19]

Controlled degradation of the extracellular matrix is essential for the invasion and metastasis of malignant tumors. In this context matrix metalloproteinases and in particular MMP2 and MMP9 are of crucial significance for tumor development and progression [20]. Indeed increasing experimental evidence indicates involvement of both enzymes at multiple steps of the metastatic process[21] It is important to note that these metalloproteinases are secreted as inactive pro- enzymes and acquire their active form extracellularly [3,22]. Taking these into account together with accumulating data demonstrating extracellular activity of HSP90 [12], we next examined the possible interaction of this molecule with the two metalloproteinases using co-immunoprecipitation experiments. Our results showed that both the α and the β isoforms of HSP90 interact with the secreted, inactive forms of MMP2 and MMP9. Interestingly, reverse co-immunoprecipitation experiments revealed that HSP90 also interacts, with the extracellularly activated forms of the two MMPs, The above observations indicate that HSP90 participates in the activation of MMP2 and MMP9. Moreover, they suggest a second level of chaperoning for this molecule associated with the mature forms of the metalloproteinases examined. Our findings are in accordance with previously reported data showing association of the secreted pool of HSP90α with MMP2 [15,23]. Moreover they provide new evidence for a further increase in the number of clients of this chaperone molecule by adding MMP9 to the list of its extracellular substrates.

We have previously demonstrated that mAb 4C5 inhibits the invasive capacity of MDAMB453 breast cancer cells by disrupting the interaction of cell surface HSP90 with the extracellular domain of HER-2, a member of the ErbB family of epidermal growth factor receptors [14]. Taking this into account together with the above mentioned findings we next investigated whether mAb 4C5 independently of its effect on HER2 has also an effect on the secretion and activation of MMP2 and MMP9. Our results showed that when the cell- impermeable mAb 4C5 [14] is added to the culture medium of MDAMB453 cells, secretion of pro-MMP2 and pro-MMP9 is not affected; however and as shown by zymography and western blot analysis, the activation of these metalloproteinases is dramatically inhibited as compared to controls where mAb 4C5 is not included in the culture medium. Moreover, co-immunoprecipitation experiments performed as described in Methods, revealed that mAb 4C5 effectively disrupts the interaction of HSP90 with MMP2 and MMP9 respectively. Our present data indicate that mAb 4C5 while not affecting secretion of the inactive forms of MMP2 and MMP9, prevents their maturation most probably by disrupting their interaction with the extracellular pool of HSP90. Similar to our results have been previously reported by Eustace et al [15] who have shown that inhibition of extracellular HSP90α decreases activation of MMP2. At this point it should be noted that preliminary experiments performed using the MDAMB231 breast cancer cells which lack the HER2 receptor, indicate not only that these cells too secrete both isoforms of HSP90 but also that MMP2 and MMP9 activation by extracellular HSP90 may be independent of the HER2/HSP90 interaction previously reported [14], since in this system as well mAb 4C5 prevented activation of these metalloproteinases (unpublished data). Nevertheless our overall observations do not exclude the possibility that in combination with HSP90 additional molecules, such as other HSPs, HSP90 co-chaperones etc may be necessary for the activation of the MMPs studied in this work. This however, needs further investigation.

We have previously demonstrated that mAb 4C5 significantly inhibits melanoma metastasis in C57BL/6 mice inoculated with B16 F10 melanoma cells, [13]. supporting accumulating data showing that antibodies or other small molecules that inhibit HSP90 can be used as anti-cancer agents [24] This prompted us to explore the anti-metastatic activity of mAb 4C5 with respect to MDAMB453 breast cancer cells. In line with the recently reported *in vitro *results [14], in this work we showed that mAb 4C5 strongly inhibits the metastatic depositions of MDAMB453 cells into the lungs of SCID mice. More specifically an 86.67% inhibition of metastatic depositions of MDAMB453 cells was observed in the mAb 4C5 treated mice as compared with control animals. It is interesting to note that in the experimental group of mice that received mAb 4C5, MDAMB453 cells were often observed stagnating on the inner surface of large pulmonary vessels, whereas such a phenomenon was very rarely detected in the control mice. Quantification of this occurrence confirmed a statistically significant difference (p < 0,001) between the two experimental groups. This remarkable observation together with the above mentioned data, tempts us to reason that mAb 4C5 exerts its activity by disrupting the interaction of extracellular HSP90 with MMP2 and MMP9 and thus preventing activation of these matrix metalloproteinases which, as is well documented [7,8], is necessary for the degradation of type IV collagen, a major constituent of the basement membrane associated with the pulmonary blood vessel. As a consequence, tumor cells remain limited to the inner surface of the vessel and their infiltration into the lung tissue is impaired. Regulation of MMP activation has been previously correlated with cancer cell extravasation. In particular Cruz-Munoz et al have reported that TIMP-3 decreases MMP2 activation which in turn limits tumor cell extravasation and subsequent colonization of the lung[25].

## Conclusions

Our present findings combined with recently reported data [13,14], reinforce and further extend the idea that extracellular chaperoning of both the α and the β isoforms of HSP90, at least in relation to cancer cell invasion and metastasis, is exerted at multiple levels such as activation of growth factor receptor as previously reported[14] and MMP2 and MMP9 activation. Our results additionally suggest that extracellular HSP90 may also chaperone the activity of the mature metalloproteinases with their corresponding substrates. However, elucidation of the precise molecular mechanisms underlying these interactions needs further investigation. Finally, our present work further demonstrates the anti-metastatic capacity of mAb 4C5, showing that it inhibits not only melanoma metastasis [13] but also the formation of metastatic breast cancer cell deposits by preventing extravasation of tumor cells and their invasion in the lung tissue.

## Methods

### Reagents

MAb4C5 was produced and characterized, as previously reported [26]. In the present study, mAb4C5 was used as concentrated serum free supernatant dialyzed in saline buffer in all experiments performed. Polyclonal antibodies specific for the α and the β isoforms of HSP90 were obtained from Chemicon. International, USA. At this point it should be noted that according to the manufacturer, the anti-HSP90β antibody shows no reactivity with HSP90α and the anti-HSP90α antibody reacts weakly with HSP90β. Polyclonal antibody recognizing both the inactivated pro-enzyme and the activated form of human MMP2 was obtained from Chemicon. International, USA. Polyclonal antibody recognizing both the inactivated pro-enzyme and the activated form of human MMP9 was obtained from Santa Cruz Biotechnology INC. Monoclonal antibody against human beta actin was obtained from Santa Cruz Biotechnology INC Polyclonal biotinylated antibodies against anti-rabbit and anti mouse immunoglobulins were obtained from Dako Cytomation Denmark A/S. Human recombinant HSP90α was a generous gift from Dr C. Prodromou, Institute of Cancer Research. Gelatin-agarose beads, used for the preclearing of the supernatants, as well as protein G and protein A-sepharose beads, used for the co-immunopercipitation experiments, were obtained from Sigma. Dulbecco's modified Eagles medium (DME), fetal bovine serum (FBS), RPMI, were obtained from Gibco, INC, USA.

### Cell cultures

The MDAMB453 human breast cancer cell line was kindly provided by Dr A.Mamalaki from the Hellenic Pasteur Institute, Athens, Greece. The cells were seeded at a density of 2×10^6 ^cells per cm^2 ^on plastic dishes as monolayers in RPMI media containing 10% FBS and supplemented with the antibiotic garamycin, (10 mg/ml) in a 5% CO2 humidified atmosphere at 37°C. When cells reached confluence, the initial medium was replaced by RPMI without FBS and the cells were left for 24 hours in starvation conditions. Subsequently they were exposed to 200 μg/ml of mAb 4C5 for 24 h. Control cultures were grown for 24 h either in RPMI medium alone or in culture medium containing 200 μg/ml of an irrelevant IgG2a monoclonal antibody named BM88 [27].

### Preparation of cell lysates and concentrated supernatants

At the termination of the cultures, cells were immediately washed twice with ice-cold PBS and lysed in 50 μl lysis buffer (137 mM NaCl, 20 mM Tris/HCl pH 7.4, 50 mM HEPES, 5 mM EDTA, 1 mM DTT, 1 %Triton, 10 %Glycerol, 200 mM Na3VO4, 0.5 mM PMSF, 5 μg/ml aprotinin, 5 μg/ml pepstatin and 50 μg/ml leupeptin). Protein lysates were quantified and equal amounts of total protein were subjected to SDS-PAGE and Western blot. Concentrated supernatants derived from MDAMB453 cells, cultured as described above, were prepared as follows: An initial volume of 20 ml of culture supernatant was collected from each experimental case (control and mAb 4C5 treated cultures). At this point it is important to underline that the supernatants were derived from the same number of cultured cells, for all experimental cases. Subsequently 100 fold concentration was performed using ultra centrifugal filter devices (10000 MWCO) obtained from Millipore TM. The concentrated supernatants were used for all the experimental procedures concerning immunoprecipitations and Western blots. Equal volumes of the concentrated supernatants were subjected to SDS-PAGE on 12% acrylamide in order to obtain the best separating conditions for the proteins of interest [28]. The analyzed proteins were transferred to nitrocellulose membranes. For Western blots, membranes were incubated for 40 min at room temperature with non-fat dry milk (5%) in TBS and were then incubated with specific primary antibodies overnight at 4°C. The membranes were washed with 0.3% BSA in TBS and incubated with horseradish peroxidase-labeled secondary antibodies for 2 h at room temperature. After washing with TBS, the bound antibody complex was detected using an ECL chemiluminescence reagent (Amersham) and XOMAT- AR film (Kodak, Pittsburgh, PA, USA) as described by the manufacturers.

### Co-immunoprecipitation

Co-immunoprecipitation was performed as previously described [29]. In brief, equal amounts of cellular lysates and equal volumes of the concentrated supernatants from control and mAb 4C5 treated cultures of MDAMB453 cells were incubated with antibodies overnight at 4°C. The immunocomplexes were then incubated for 2 h at room temperature with protein G-Sepharose or protein A-Sepharose (for the mAb 4C5 treated cultures) and washed 3 times with lysis buffer. At this point it should be noted that mAb 4C5 does not bind to protein A Sepharose (unpublished observations) thus it's presence did not interfere with the immunoprecipitation process. Bound proteins were analyzed by gel electrophoresis and Western blot as described above.

### Isolation of metalloproteinases

Isolation of the MMPs from the other proteins present in the concentrated supernatants was performed as previously described[30]. Briefly gelatin-Agarose beads were used in order to form two columns. The concentrated supernatants of the control and mAb 4C5 treated cultures were loaded on the two columns and washed with an equilibration buffer containing 0.05Μ Tris-HCl pH:7,5/0.5 M NaCl, and 0.1% Triton X-100. Gelatine bound MMPs were eluted with equilibration buffer containing 1 M NaCl and 5% (v/v) dimethyl sulphoxide.

### Zymographs

Equal volumes of isolated MMPs derived from control and mAb 4C5 treated cultures of MDAMB453 cells, were denatured and analyzed by gel electrophoresis in a 10% SDS-polyacrylamide gel containing 0.1% (w/v) gelatin. The gel was incubated at room temperature for 2 h in the presence of 2.5% Triton x-100 and subsequently at 37°C over night in a buffer containing 10 mM CaCl2, 0.15 M NaCl, and 50 mM Tris (pH 7.5). The gel was then stained for protein with 0.5% (w/v) Coomassie and photographed on a light box. Proteolysis was detected as a white zone in a dark field. MMPs (proenzymes and active forms) isolated as previously described [30] from extracts of interface tissue from loose hip arthroplasty endoprostheses were used as markers (kindly provided by Dr Aletras).

### Assay of MDAMB453 cell metastatic deposit formation in the lung tissue

SCID mice were originally purchased from Jackson Laboratory, bred and maintained under specific pathogen free conditions at the Experimental Animal Unit of the Hellenic Pasteur Institute. All of the experiments with animals were done in accordance with the guidelines approved by the Ethical Committee of the Hellenic Pasteur Institute. The *in vivo *metastatic deposit formation assay, was performed as previously described [31,17] Briefly, cultured MDAMB453 cells were pre-incubated with DiI for 1 hour, washed twice with PBS, trypsinized and made up to the cell density of 10^6^/300 μL in PBS in the absence or presence of 100 μg/ml of an irrelevant antibody named of mAb BM88[27] or 100 μg/ml of mAb 4C5. Twenty 8- 10-week-old female SCID mice were injected through the tail vein with 0.3 ml of the above cell preparations. The animals were divided into two equal groups: the control group injected with cells dialysed in PBS or the irrelevant antibody, mAb BM88 and the mAb 4C5 treated group. The animals were euthanized 24 hours later and with the use of a peristaltic pump connected to the left ventricle of the heart, their lungs were completely washed from the remaining blood, by pumping 200 ml of saline buffer. This procedure ensures that all cancer cells which are not attached, either on the inner surface of the blood vessels or on the lung tissue, are removed. Finally the lungs were perfused with 4% formalin solution and then embedded in OCT solution in order to perform cryosections. Each lung was sectioned with the cryotome and each section was counter stained with Dapi and visualised with a confocal microscope. In ten randomly chosen slides covering the whole of the lung tissue the MDAMB453 cells were calculated. The same experiment was performed twice with similar results.

### Evaluation of tumor cell retention in the pulmonary vessels

In the above mentioned lung sections the total number of blood vessels was calculated and MDAMB453 stagnating cells were visualized and compared between the control and the mAb 4C5 treated animals.

### Statistical analysis

For the experimental groups that were statistically analyzed for differences, Student's t test was used, in which *p *< 0.05 was defined as statistically significant.

## Authors' contributions

DS participated in the design of the study carried out, part of the i.p. experiments, the in vivo experiments and the statistical analysis. Moreover he participated in the drafting of the manuscript. AH performed part of the i. p. experiments and the zymography. EP conceived of the study, oversaw the experimental design and wrote the final draft of the manuscript. All authors read and approved the final manuscript.

## Supplementary Material

Additional file 1 **Additional figure showing that the antibody against HSP90β does not recognize the HSP90α isoform**. The western blot analysis of human recombinant HSP90α and total cell lysates (positive control) derived from MDAMB453 cell cultures using anti-HSP90α (positive control) and anti-HSP90β antibodies. Click here for file

Additional file 2 **Additional figure legend**. A file containing the legend of the additional figure. Click here for file
