# Supplementary figures and images for: Monoclonal antibody 4C5 prevents activation of MMP2 and MMP9 by disrupting their interaction with extracellular HSP90 and inhibits formation of metastatic breast cancer cell deposits
Source: BMC Cell Biol. 2010 Jul 5;11:51. doi: 10.1186/1471-2121-11-51 (PMC2914660; doi:10.1186/1471-2121-11-51)

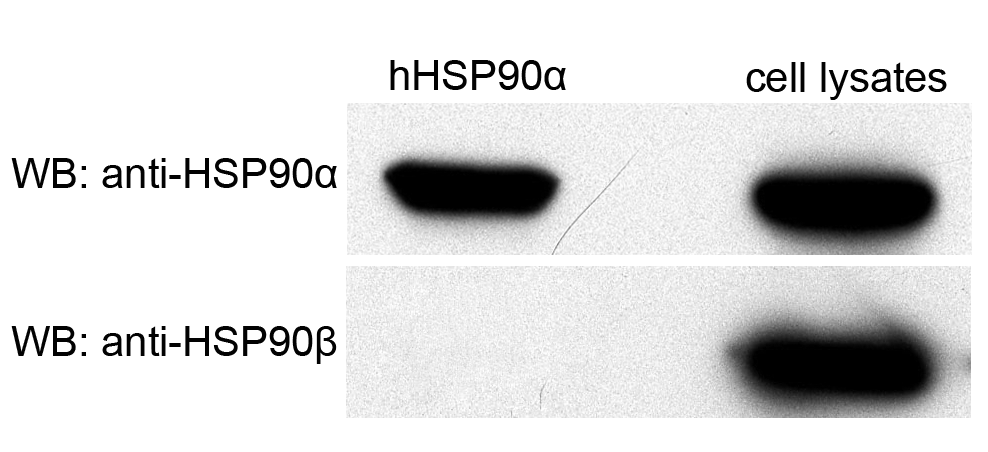

Supplement: Additional file 1 — Additional figure showing that the antibody against HSP90β does not recognize the HSP90α isoform. The western blot analysis of human recombinant HSP90α and total cell lysates (positive control) derived from MDAMB453 cell cultures using anti-HSP90α (positive control) and anti-HSP90β antibodies. [file 1471-2121-11-51-S1.TIFF]
