# Supplementary material for: Monoclonal antibody 4C5 prevents activation of MMP2 and MMP9 by disrupting their interaction with extracellular HSP90 and inhibits formation of metastatic breast cancer cell deposits
Source: BMC Cell Biol. 2010 Jul 5;11:51. doi: 10.1186/1471-2121-11-51 (PMC2914660; doi:10.1186/1471-2121-11-51)
Supplement: Additional file 2 — Additional figure legend. A file containing the legend of the additional figure. [file 1471-2121-11-51-S2.DOC]

**Additional Figure Legend.**

**Antibody against HSP90β does not recognize the HSP90α isoform:** Human recombinant HSP90α and total cell lysates (positive control) derived from MDAMB453 cell cultures were analyzed by Western blot using anti-HSP90α (positive control) and anti-HSP90β antibodies. The absence of immunostaining observed using anti-HSP90β on the recombinant HSP90α protein confirms the specificity of the antibody.
